# Acquired Resistance to Afatinib Mediated by EGFR T790M in Lung Adenocarcinoma Patients Harboring EGFR-KDD: A Case Report and Literature Review

**DOI:** 10.3390/curroncol33040214

**Published:** 2026-04-14

**Authors:** Qian Liu, Lu Lv, Guanchao Pang, Pingli Wang

**Affiliations:** Department of Respiratory and Critical Care Medicine, The Second Affiliated Hospital of Zhejiang University School of Medicine, Hangzhou 310009, China; qianl@zju.edu.cn (Q.L.); lucyannabelle@zju.edu.cn (L.L.); pangguanchao@zju.edu.cn (G.P.)

**Keywords:** EGFR-KDD, lung adenocarcinoma, tyrosine kinase inhibitor, EGFR T790M, acquired resistance

## Abstract

Epidermal growth factor receptor (EGFR)-kinase domain duplication (KDD) represents an atypical mutation distinct from classical EGFR mutations in lung adenocarcinoma (LUAD). Although first- and second-generation EGFR-tyrosine kinase inhibitors (TKIs) have demonstrated clinical activity in EGFR-KDD, the mechanisms of acquired resistance in this setting remain poorly characterized. Herein, we present a LUAD patient with EGFR-KDD who achieved a sustained initial response to afatinib lasting 67 months before developing acquired resistance. Re-biopsy with next-generation sequencing (NGS) uncovered EGFR T790M, accompanied by EGFR amplification and EGFR M766T. The patient was switched to firmonertinib, with subsequent tumor regression. We reviewed published EGFR-KDD cases that had both acquired resistance to first- or second-generation EGFR-TKIs and corresponding repeat biopsy findings. Five of the eleven cases harbored EGFR T790M, yielding a prevalence of 45%, which is similar to that seen in classical EGFR mutations. This case suggests that EGFR T790M mediates acquired resistance to first- and second-generation EGFR-TKIs in EGFR-KDD, mirroring the resistance pattern observed in classical EGFR mutations.

## Simple Summary

Epidermal growth factor receptor (EGFR)-kinase domain duplication (KDD) represents an uncommon genetic alteration identified in a minority of patients with lung cancer, but the mechanisms underlying resistance to first- and second-generation EGFR-tyrosine kinase inhibitors (TKIs) remain unclear. We report a patient with EGFR-KDD who developed an EGFR T790M mutation after disease progression on afatinib and achieved a second remission following a switch to firmonertinib. We also conducted a literature review of EGFR-KDD cases to further refine treatment strategies for patients with this rare mutation.

## 1. Introduction

Lung cancer is the leading cause of cancer-related mortality worldwide and ranks second in cancer incidence [1]. Non-small-cell lung cancer (NSCLC) comprises the largest proportion of lung cancer, among which lung adenocarcinoma (LUAD) represents roughly half of the total diagnosed cases. Improvements in next-generation sequencing (NGS) have shifted tumor molecular subtyping from conventional pathological morphology toward precision genomics. Targeted therapies against genetic mutations have exhibited notable clinical efficacy in patients with advanced NSCLC, substantially extending both PFS and OS. These treatments have shifted the previous chemotherapy-dominated paradigm, greatly improving both survival outcomes and quality of life. Accordingly, comprehensive exploration of actionable therapeutic targets in lung cancer is essential for advancing precision treatment strategies and facilitating individualized care, with substantial implications for both clinical practice and scientific research [2].

Epidermal growth factor receptor (EGFR) mutations are frequently identified actionable mutations in NSCLC, occurring in at least 50% of Asian patients [3]. Beyond classical EGFR mutations such as exon 19 deletions and the L858R point mutation, the EGFR gene can also harbor kinase domain duplication (KDD), which was first reported in lung cancer in 2015 [4,5]. EGFR-KDD typically involves in-frame tandem duplications spanning exons 18–25, with rare cases involving exons 14–26 or 17–25 [6]. This large genomic rearrangement results in duplication of the EGFR kinase domain at the protein level, forming an intramolecular dimer that leads to sustained activation of downstream signaling pathways [7]. A large multicenter retrospective study identified 13 cases of EGFR-KDD among 10,759 NSCLC patients, with a frequency of approximately 0.12% in the overall cohort and 0.24% in those with EGFR mutations [6]. Multiple studies have shown that first- and second-generation EGFR-TKIs are effective in EGFR-KDD. However, due to the low incidence of this mutation, the mechanisms of acquired resistance to first- and second-generation EGFR-TKIs remain poorly understood. Clarifying these resistance mechanisms is therefore essential to enhance clinical understanding and inform treatment decisions for patients with EGFR-KDD.

Here we report a LUAD patient harboring EGFR-KDD who developed an EGFR T790M mutation upon resistance to afatinib. We reviewed all previously reported cases of EGFR-KDD with acquired resistance to first- and second-generation EGFR-TKIs and available repeat biopsy data, summarized the genetic alterations identified upon resistance, and analyzed the mechanisms of acquired resistance in EGFR-KDD.

## 2. Case Presentation

**Figure 1:**
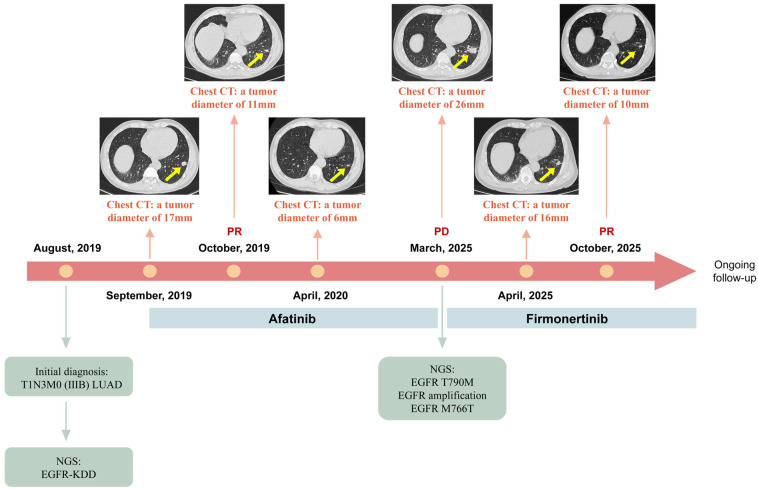
A summary of the disease course of the patient. LUAD, lung adenocarcinoma; EGFR, epidermal growth factor receptor; KDD, kinase domain duplication; CT, computed tomography; PR, partial response; PD, progressive disease. The yellow arrows indicate the lesions.

We present a follow-up case report on a 66-year-old male LUAD patient harboring EGFR-KDD, previously documented for its durable response to afatinib. The details regarding his initial clinical presentation, diagnosis and management have been previously described in the earlier report [8]. In brief, he was a smoker (15 cigarettes/day for 40 years) and was diagnosed with T1N3M0 (IIIB) LUAD in August 2019. Baseline NGS of the primary tumor sample identified an EGFR-KDD involving exons 18–25 (CN: 4.6), with no other classical EGFR mutations detected. Figure 1 illustrates the disease course of the patient. The patient received afatinib therapy (30 mg once daily) in September 2019 and achieved a durable response, with a 64.7% reduction in tumor diameter. Following slight radiological progression on chest CT in March 2021, the patient received intensity-modulated radiotherapy (IMRT) with 6 MV X-rays targeted at the left lower lobe lesion (50 Gy in 5 fractions, delivered to PTV D95) while continuing oral afatinib, with a mean lung dose of 3.6 Gy, V20 of 3.6%, and V5 of 22%.

The durable response continued until March 2025, when CT imaging showed marked tumor enlargement within the irradiation field and CT-guided biopsy confirmed disease progression. Genetic testing of the tumor tissue revealed the emergence of EGFR T790M (VAF: 2.42%), accompanied by EGFR amplification (CN: 2.8) and EGFR M766T (VAF: 1.18%). The patient was subsequently switched to the third-generation EGFR-TKI firmonertinib and again achieved a favorable response, with a 61.5% reduction in tumor diameter by October 2025. No adverse events occurred during the treatment course. The patient continues to receive firmonertinib at the time of this report.

## 3. Discussion

Approximately half of acquired resistance cases to first- and second-generation EGFR-TKIs in classical EGFR-mutant NSCLC are attributable to the EGFR T790M mutation. EGFR T790M enhances the ATP-binding affinity of EGFR, resulting from the replacement of threonine by methionine within the tyrosine kinase region [9]. Third-generation EGFR-TKIs circumvent EGFR T790M-mediated resistance through covalent engagement with the mutant EGFR protein. In the FLAURA trial, osimertinib significantly improved PFS (18.9 vs. 10.2 months) and OS (38.6 vs. 31.8 months) compared to first-generation TKIs, establishing it as the first-line standard therapy for advanced EGFR-mutant NSCLC [10]. Although the role of EGFR T790M in classical EGFR mutations has been extensively studied, whether it plays a similar role in EGFR-KDD remains unclear, largely due to the low incidence of EGFR-KDD and the limited number of reported cases.

**Figure 2:**
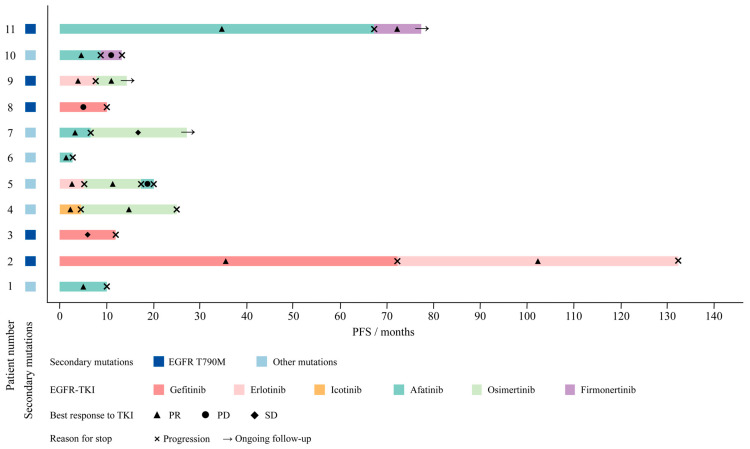
Swimmer plot of PFS and secondary mutations in patients treated with different EGFR-TKIs across the 11 cases. PFS, progression-free survival; PR, partial response; PD, progressive disease; SD, stable disease.

We reviewed all previously reported cases of EGFR-KDD with acquired resistance to first- and second-generation EGFR-TKIs and available repeat biopsy data (Table 1, Figure 2). Among the 11 cases identified, the EGFR T790M mutation was present after resistance to first- and second-generation EGFR-TKIs in five, including our case. Based on our literature review, the incidence of EGFR T790M in EGFR-KDD was approximately 45%, a rate comparable to that observed in classical EGFR mutations. Baik et al. reported a 45-year-old female who developed resistance after sequential treatment with gefitinib and erlotinib, with repeat genetic testing revealing EGFR T790M [5]. A multicenter retrospective study identified an EGFR-KDD patient who progressed after 11 months of gefitinib therapy and was found to harbor EGFR T790M [6]. Lai et al. also reported the emergence of EGFR T790M in an EGFR-KDD patient who progressed after 10 months of gefitinib treatment [11]. Parallel to our case, Lee et al. reported a patient harboring EGFR-KDD who, following resistance to erlotinib and the emergence of EGFR T790M, achieved a partial response after switching to the third-generation EGFR-TKI osimertinib. Notably, they further performed in vitro experiments demonstrating that EGFR T790M in either one or both kinase domains mediates resistance to first- and second-generation EGFR-TKIs in EGFR-KDD, whereas sensitivity to third-generation agents is preserved [12]. Therefore, while the NGS testing employed in our case was unable to distinguish whether EGFR T790M occurred in one or both copies of the duplicated kinase domains, the role of EGFR T790M in mediating resistance to afatinib while retaining sensitivity to firmonertinib is supported. Future application of more accurate detection techniques would be valuable for resolving this issue in clinical specimens.

Given that the in-frame tandem duplication in EGFR-KDD confers structural differences from classical EGFR mutations, it remains unclear whether EGFR T790M plays an analogous role in acquired resistance to first- and second-generation EGFR-TKIs in EGFR-KDD. A recent study shows that regardless of whether EGFR harbors classical mutations or KDD, its kinase domains require asymmetric dimerization for full activation. Despite the tandem duplication, the two tyrosine kinase domains (TKDs) in EGFR-KDD associate only weakly and transiently. This dynamic equilibrium renders the oncogenic activity of EGFR-KDD readily regulable [18]. EGFR T790M confers resistance to first- and second-generation EGFR-TKIs primarily by increasing ATP affinity, a mechanism independent of the EGFR mutation format. The asymmetric dimerization in EGFR-KDD does not alter the biochemical properties of the ATP-binding pocket. Thus, when EGFR T790M emerges in KDD, first- and second-generation EGFR-TKIs lose efficacy due to heightened ATP competition, while third-generation EGFR-TKIs overcome this via covalent binding to Cys797, effectively inhibiting the kinase whether in loose KDD dimers or classical mutant forms. This suggests that EGFR T790M-mediated resistance to first- and second-generation EGFR-TKIs in EGFR-KDD shares a conserved mechanism with classical EGFR mutations.

Repeat genetic testing revealed not only EGFR T790M but also EGFR M766T and EGFR amplification in our case. EGFR amplification is a common resistance mechanism in classical EGFR mutations, and EGFR M766T has also been reported to confer resistance to gefitinib and erlotinib [19]. This pattern of co-occurring secondary mutations was also observed in the majority of the other 10 cases we reviewed. This suggests that acquired resistance to first- and second-generation EGFR-TKIs in EGFR-KDD may be mediated by multiple mechanisms, with EGFR T790M representing only one of them. The coexistence of multiple resistance alterations in EGFR-KDD likely reflects intratumoral heterogeneity under therapeutic selection pressure, a phenomenon well documented in classical EGFR mutations.

## 4. Conclusions

This case adds to the understanding of acquired resistance mechanisms to first- and second-generation EGFR-TKIs in EGFR-KDD. In our case, the emergence of EGFR T790M following afatinib resistance and the subsequent response to firmonertinib closely mirror the established treatment paradigm observed in classical EGFR mutations. Supporting this clinical observation, our literature review revealed that the incidence of EGFR T790M in EGFR-KDD is comparable to that observed in classical EGFR mutations. Mechanistically, structural analyses suggest that the tandem duplication in EGFR-KDD does not impair the EGFR T790M-mediated enhancement of ATP affinity. Furthermore, we noted that concurrent resistance mutations frequently arise in EGFR-KDD, reflecting the heterogeneity well documented in classical EGFR mutations. Collectively, these findings demonstrate that EGFR T790M mediates resistance to first- and second-generation EGFR-TKIs in EGFR-KDD through a mechanism analogous to its role in classical EGFR mutations.

The rarity of EGFR-KDD precludes large-scale prospective studies. Our report aims to provide therapeutic insights for patients with EGFR-KDD and advocates for repeat biopsy with NGS upon acquired resistance to guide timely treatment adjustments. Continued accumulation of cases with comprehensive clinical course will be essential to fully elucidate the spectrum of resistance mechanisms and to optimize treatment strategies in this understudied patient population.

## Figures and Tables

**Table 1:** Clinical characteristics and outcomes of lung cancer patients harboring EGFR-KDD with acquired resistance to first- and second-generation EGFR-TKIs and available repeat biopsy data in previous studies.

No.	Publication	Age	Gender/Ethnicity	Stage	EGFR-TKI/Generation No.	Best Response to TKI	PFS	Secondary Mutations After TKI Resistance
1	Gallant et al. 2015 [4]	33	Male/American	IV	Afatinib/2nd generation	PR	10 mo.	EGFR amplification
2	Baik et al. 2015 [5]	45	Female/American	NA	Gefitinib/1st generation Erlotinib/1st generation	PR PR	6 yr. 5 yr.	EGFR T790M CTNNB1 S37C
3	Wang et al. 2019 [6]	60	Female/Chinese	IV	Gefitinib/1st generation	SD	11 mo.	EGFR T790M EGFR amplification
4	Li et al. 2020 [13]	45	Male/NA	IIIA	Icotinib/1st generation Osimertinib/3rd generation	PR PR	4 mo. 21 mo.	RELN G1774E
5	Hirokawa et al., 2021 [14]	45	Female/Japanese	NA	Erlotinib/1st generation Osimertinib/3rd generation Afatinib/2nd generation	PR PR PD	133 day 14.5 mo. 1 mo.	TP53 R65fs*58 FGFR4 R248Q ATRX R498G VEGFA S186F
6	Kim et al. 2021 [15]	50	Male/African- American	IV	Afatinib/2nd generation Osimertinib/3rd generation	PR NR	2 mo. NR	EGFR amplification FANCD truncation
7	Zhang et al. 2022 [16]	44	Male/Chinese	IVA	Afatinib/2nd generation Osimertinib/3rd generation	PR SD	7 mo. 20.5 mo.	EGFR amplification
8	Lai et al. 2022 [11]	NA	NA	NA	Gefitinib/1st generation	PD	10 mo.	EGFR T790M
9	Lee et al. 2022 [12]	56	Male/NA	IV	Erlotinib/1st generation Osimertinib/3rd generation	PR PR	8 mo. 7 mo.	EGFR T790M
10	Chen et al. 2025 [17]	71	Female/Chinese	IVB	Afatinib/2nd generation Firmonertinib (+Crizotinib) /3rd generation	PR PD	9 mo. 4 mo.	TP53 exon c688_764del MET-LGR
11	our case	66	Male/Chinese	IIIB	Afatinib/2nd generation Firmonertinib/3rd generation	PR PR	67 mo. 10 mo., NR	EGFR T790M EGFR M766T EGFR amplification

EGFR, epidermal growth factor receptor; KDD, kinase domain duplication; TKI, tyrosine kinase inhibitor; No., number; LUAD, lung adenocarcinoma; PFS, progression-free survival; NA, not available; PR, partial response; PD, progressive disease; SD, stable disease; NR, not reached; mo., month; yr., year.

## Data Availability

The original contributions presented in this study are included in the article. Further inquiries can be directed to the corresponding author.

## Abbreviations

The following abbreviations are used in this manuscript:

EGFR	Epidermal growth factor receptor
KDD	Kinase domain duplication
NSCLC	Non-small-cell lung cancer
LUAD	Lung adenocarcinoma
PFS	Progression-free survival
OS	Overall survival
TKI	Tyrosine kinase inhibitor
NGS	Next-generation sequencing
CT	Computed tomography
No.	Number
NA	Not available
PR	Partial response
PD	Progressive disease
SD	Stable disease
NR	Not reached
TKDs	Tyrosine kinase domains
